# Early maladaptive schemas in female patients with migraine and tension-type headache

**DOI:** 10.1038/s41598-024-53816-4

**Published:** 2024-02-12

**Authors:** Gözde Yontar, Esen Ozgan

**Affiliations:** 1 Psychiatry Clinic, Samsun Training and Research Hospital, Körfez Mah. 5013. Sok. 2/7, Atakum, Samsun, Turkey; 2 Neurology Clinic, Samsun Training and Research Hospital, Samsun, Turkey

**Keywords:** Migraine, Tension-type headache, Early maladaptive schemas, Human behaviour, Headache

## Abstract

Chronic headache is a serious clinical problem in women which psychological factors play major role and requires an approach with bio-psycho-social integrity. Psychiatric comorbidities such as anxiety disorder and major depression are frequent. Young described Early Maladaptive Schemas (EMS) as maladaptive and dysfunctional patterns that appear due to unpleasant situations occurred between patient and people who were important to him. EMS affect perception, emotions, thoughts and behaviors that makes a basis for many disorders. EMS are found to be related with depression, anxiety and somatization within recent literature. In literature, chronic pain and migraine’s relationship with EMS were studied before in a few separate articles. However, there is a lack of data that compares the relationship between EMS and clearly distinguished headache types: migraine (MH) and tension-type headache (TTH) especially in female patients who are more prone to somatic complaints/findings. Our study directly compared three groups, migraine and tension type headache and healthy controls. 150 female patients with chronic headache were enrolled for study in consecutive fashion. Exclusion criteria were as follows: age < 18 or > 60 years, male gender, existence of comorbidity affecting central nervous system, headache due to drug/substance abuse, previous diagnosis of migraine with aura, previous diagnosis of psychotic disease, mental retardation, cognitive disorders, being in delirium state. Patients were grouped into two categories according to type of headache and a third control group. All patients were evaluated with Young Schema Questionnaire (YSQ) and their scores were noted and categorized in related schema domains. Sociodemographic data were comparable among groups. However, there were significant differences in terms of previous psychiatric diagnosis and psychiatric drug usage. When we compared YSQ scores, groups differed in many schema categories. MH group showed significantly higher scores in dependency/incompetency, unrelenting standards and punitiveness schemas when compared to remaining two groups. TTH group had significantly more points in emotional deprivation, vulnerability to harm or illness schemas among all groups. On the other hand, control group had significantly higher scores in insufficient self-discipline and entitlement/grandiosity schemas when compared to both MH and TTH groups. Presence of EMS in female patients with headache significantly differed from control group. Types of EMS were also significantly different between patients with MH and TTH among this whole headache group. We suggest that a comprehensive psychotherapeutic approach that targets to improve coping methods for distinct EMS in distinct headache types would provide critical aid to general treatment of headaches especially in resistant cases.

## Introduction

Chronic pain is a serious clinical problem, in which psychological factors play a major role^1^. Therefore, patients with chronic pain should be approached using a method with biopsychosocial integrity. Headaches are one of the most common types of chronic pain. Psychological distress may contribute to the etiology of headaches or may be merely accompanied by existing headache diseases^2^. Studies have shown that psychiatric comorbidities such as anxiety disorders and major depression are frequent in these patients. It is well known that these psychiatric diseases are mostly encountered in women^3^. Women are more commonly admitted to headache clinics than men^3^.

Early maladaptive schemas (EMS) are related to depression, anxiety, and somatization with in recent literature^4,5^. Young et al.^6^ described EMS as maladaptive and dysfunctional patterns that appear because of unpleasant situations that occur between patients and people who are important to them. These patterns are resistant to change; therefore, they cannot change according to conditions. This affects perception, emotions, thoughts, and behaviors that form the basis of many disorders.

Somatization plays an important role in schema theory. Headaches also have unignorable underlying somatic mechanisms^7^. EMS are associated with chronic pain syndrome and migraine^8,9^. However, the relationship between different types of headaches and EMS remains unclear. The aim of our study was to investigate EMS for two major headache types, migraine and tension-type headache, in female patients who are more susceptible to somatization.

Headache is not only a common public health problem but also a disease that is related to many psychopathologies, which is why it negatively affects individuals’ functionality in daily life, social, and occupational environments. The etiology of this common complaint is classified into two groups: primary and secondary headache. Primary headache is responsible for almost 95% of all headache complaints, and 90% of this category consists of tension-type, migraine, and cluster-type headaches^10^. Epidemiological studies have shown that migraines are the most common type of headache. Fifteen percent of the world’s population suffers from migraine. The overall life risk of migraine is 43% in women and 18% in men. It is difficult to underestimate the economic effects of this widespread disease, such as workforce losses and healthcare costs. With these restrictive effects on the social lives of patients, migraines have been extensively studied. However, the etiology of primary headaches remains unclear. Several studies have been conducted on the pathogenesis of primary headaches. Chronic migraine and chronic tension-type headache are devoid of the acute stimulating effects of physiological nutrition and cause permanent anatomical, pharmacological, and physiological changes in the central nervous system and pain receptors. Chronic headaches are usually associated with other psychiatric diseases at a rate of 30–100%. The incidence of depression in these patients is usually higher than that in the remaining population. The severity of depression increases with an increase in chronic headache symptoms. Anxiety disorders were also common in this group. Moreover, 31–51% of patients with chronic headaches are diagnosed with at least one type of personality disorder^11^. Other common symptoms in patients with chronic headache include tiredness and irritability. Anger and hopelessness negatively affect headaches. These patients develop negative, unrealistic thoughts and beliefs in addition to pre-existing problems. They focused on odd events and stressors caused by emotional stress in their lives. Consequently, a vicious cycle of stress and pain may develop. Negative thoughts and emotional stress may increase the degree of pain by increasing muscle tension.

Another concept associated with psychological disorders such as depression, anxiety, and somatization are EMS, which occupy a respectable place in literature^4,5,8,9^. Young et al.^12^ explained that schemas were maladaptive and nonfunctional patterns that became complicated in time and arose from negative childhood experiences between an individual and people important to him/her. In the first few years of life and early childhood, failure to meet basic needs, especially in relation to parents and other social environments, plays a major role in the creation of EMS. Adverse interactions in early childhood define the quality of interactions between individuals and other people^12,13^. Young et al.^12^ asserted that due to its change-resistant nature, EMS created a basis for many disorders that affect perception, emotion, thought, and behavior. Studies have suggested a relationship between these disorders and EMS, especially in disorders related to chronic pain syndrome, such as depression and anxiety^4,5,8,14,15^. This suggests a similar relationship between EMS and chronic headache.

A study that investigated the relationship between early maladaptive schemas and chronic pain syndrome^8^ included 271 patients with chronic pain syndrome. A significant relationship was observed between early maladaptive and chronic pain syndromes. Furthermore, 58.3% of the study patients had high scores on the Young Schema Scale, and patients with higher scores showed a significantly higher intensity and duration of pain. In another study^5^, patients with chronic pain syndrome had significantly higher total schema points than pain-free patients in terms of EMS. The patient had two schema structures. Most patients in the first group experienced defectiveness/shame, failure, emotional deprivation, emotional inhibition, social isolation and alienation, and subjugation, whereas the second group experienced self-sacrifice, approval-seeking/recognition-seeking, and punitiveness.

Somatization is another disorder that is strongly associated with chronic headache^7^. It is known that emotions that patients cannot cope with are suppressed and surfaced as physical signs and symptoms, mostly pain. Somatization plays an important role in schema theory. According to schema theory, somatization is a defense mechanism that overcomes the burden caused by EMS. The Young Rygh Avoidance Scale was developed to identify coping mechanisms for early maladaptive schemas. Somatization is a sub-dimension of this scale as an unhealthy coping strategy. It is postulated that people who use this coping mechanism tend to avoid negative experiences and emotions. Because they cannot cope with negative experiences in a functional manner, they externalize them as several diseases with no physiological source in the body^16^. Early maladaptive schemas and chronic headaches are associated with somatization, which is thought to be another manifestation of the relationship between these two diseases.

Shahamat et al.^4^ investigated the relationship of early maladaptive schemas with somatization, depression, and anxiety. They found that not only schemas were correlated with these three parameters, but defectiveness/shame also showed the most significant relationship with other schemas. Another study^17^ found that all schemata were significantly correlated with these three parameters. Social Isolation/alienation, mistrust/abuse, abandonment/instability, negativity/pessimism, and failure were the schemata that showed the most significant relationships. In their study Henker et al.^18^ compared patients who were diagnosed with somatoform disorder according to the DSM-IV with a control group and demonstrated that high somatization was associated with generally higher schema activation.

Many studies have addressed the psychopathology of schemata^12,17,19^. Similarly, there are some studies on the psychological signs of chronic headaches (migraine and tension). In the literature, there are studies sought to examine the relationship between chronic pain and/or headache and early maladaptive schemas. In these articles female gender has a remarkable incidence of certain types of EMS namely unrelenting standards and self-punishment^9^. In addition, patients who suffered from migraine in both genders had significantly higher incidence of these two EMS. Nonetheless, patients with chronic pain showed significantly higher YSQ values of emotional deprivation, vulnerability to harm and negativity/pessimism^14^. We sought to conduct a study in order to clarify if certain types of EMS exist in both headache and female gender situations. In literature, chronic pain and migraine’s relationship with EMS were studied before in a few separate articles^5,8,9^. However, to our knowledge, there is a lack of data that compares the relationship between EMS and clearly separated headache types: migraine (MH) and tension-type headache (TTH) especially in female patients who are more prone to somatic complaints/findings^3^. Our study directly compared three groups, migraine and tension type headache and healthy controls.

## Method

### Patient enrollment

Female patients who were admitted to Neurology and Psychiatry outpatient clinics in Samsun Training and Research Hospital with complaints of headache were enrolled in a consecutive study between June 2022 and December 2022. Authors decided to conduct this study during summer and autumn season of year in order to avoid bias for patient applications to hospital like students who came from other cities, seasonal workers, especially people who apply for health reports to start a new job. This condition mostly affects control group recruitment. The exclusion criteria were: age < 18 or > 60 years, male sex, presence of comorbidities affecting the central nervous system, headache due to drug/substance abuse, previous diagnosis of migraine with aura, headache duration is less than at least 3 months during preceding twelve months, previous diagnosis of psychotic disease, mental retardation, cognitive disorders, and delirium. Age range was decided after careful scanning of literature for articles about similar subjects that utilize Young Schema Questionnaire. Also, articles and post doctorate thesis which were conducted in Turkish population using Turkish version of YSQ used same age-range. According to these findings authors decided to use current age-range would be appropriate. The patients were grouped into two categories according to headache type: migraine and tension-type headache. Types of headaches were determined by neurological evaluation, which was in compliance with The International Classification of Headache Disorders, 3rd edition (IHCD-3)^10^. Following criteria were used for chronic migraine diagnosis (1.3 code in IHCD-3) according to guideline:

Description: Headache occurring on 15 or more days/month for more than three months, which, on at least eight days/month, has the features of migraine headache.

Diagnostic criteria:

A. Headache (migraine-like or tension-type-like) on ≥ 15 days/month for > 3 months, and fulfilling criteria B and C
B. Occurring in a patient who has had at least five attacks fulfilling criteria B–D for migraine without aura and/or criteria B and C for migraine with aura (migraine with aura automatically wase excluded from study)
C. On ≥ 8 days/month for > 3 months, fulfilling any of the following:
  1. Criteria C and D for migraine without aura
  2. Criteria B and C for migraine with aura (migraine with aura automatically wase excluded from study)
  3. Believed by the patient to be migraine at onset and relieved by a triptan or ergot derivative
D. Not better accounted for by another ICHD-3 diagnosis.

Following criteria were used for chronic tension type headache diagnosis (2.3 code in IHCD-3) according to guideline:

Description: A disorder evolving from frequent episodic tension-type headache, with daily or very frequent episodes of headache, typically bilateral, pressing or tightening in quality and of mild to moderate intensity, lasting hours to days, or unremitting. The pain does not worsen with routine physical activity, but may be associated with mild nausea, photophobia or phonophobia.

Diagnostic criteria:

A. Headache occurring on ≥ 15 days/month on average for > 3 months (≥ 180 days/year), fulfilling criteria B–D
B. Lasting hours to days, or unremitting
C. At least two of the following four characteristics:
  1. bilateral location
  2. pressing or tightening (non-pulsating) quality
  3. mild or moderate intensity
  4. not aggravated by routine physical activity such as walking or climbing stairs
D. Both of the following:
  1. No more than one of photophobia, phonophobia or mild nausea
  2. Neither moderate or severe nausea nor vomiting
E. Not better accounted for by another ICHD-3 diagnosis.

The power analysis was conducted using the G*Power v3.1.9.6 program. In the reference study^16^, when considering the Dependence/Incompetence (DI) results in Table 2, with a confidence level of 95% (1 − α), a test power of 95% (1 − β), and an effect size (f) of 0.342, the minimum number of patients to be included in the study is determined to be 46 in each group, totaling 138 patients. After the evaluation, consecutive fifty patients were enrolled in each group (100 patients in total). Age-sex matched control group patients were enrolled from a group of volunteers who were admitted to the hospital for routine health examinations for different causes, job applications, university registrations, and so on. The same exclusion criteria were applied to this group, and volunteers who were presumed to have created significant differences between the control and patient groups in terms of marital status, education degree, and economic level were excluded; the remaining 50 volunteers were included in the study.

This study was conducted in accordance with the principles of the Declaration of Helsinki. All participants were expected to give signed informed consent. The local ethics committee approved the study protocol (Samsun Eğitim ve Araştırma Hastanesi Girişimsel Olmayan Klinik Araştırmalar Etik Kurulu, protocol code: GOKA/2021/14/12).

### Sociodemographic data form

All participants were expected to fill a form prepared by the researcher and gather data about age, gender, marital status, occupation, education degree, financial status, previous diagnoses of psychiatric illness, and use of psychiatric drugs (Table 1).

**Table 1:** Young schema questionnaire, schema domains and early maladaptive schemas.

Disconnection and rejection	∙ Fear of abandonment ∙ Mistrust ∙ Emotional deprivation ∙ Defectiveness/shame ∙ Social isolation/alienation
Impaired autonomy and performance	∙ Dependency/incompetency ∙ Vulnerability to harm or illness ∙ Enmeshment ∙ Failure
Impaired limits	∙ Entitlement/grandiosity ∙ Insufficient self-discipline
Orientation to the other	∙ Subjugation ∙ Self-sacrifice ∙ Recognition-seeking
Hypervigilance and inhibition	∙ Negativity/pessimism ∙ Emotional inhibition ∙ Unrelenting standards ∙ Punitiveness

### Specifications of the young schema questionnaire: short form 3 (YSQ-S3)

The EMS questionnaire used in this study was developed by Jeffrey Young in 2003^11^ and adapted to Turkish by Soygut et al. in 2009^12^. Originally, the YSQ^13^ consisted of 205 statements that evaluated 16 schemas to identify early life maladaptive schemas. Because of the length of the original questionnaire, it was considered time consuming and less useful. The YSQ-S3 was used instead of the original version. First, 75 statements were included to evaluate 15 schemas. Young identified 18 EMS organized into five domains (disconnection/rejection, impaired autonomy, impaired limits, orientation to the other, and hypervigilance and inhibition)^11^, which, when impaired, lead to dysfunctional schemas. Later, it reached a final form and become a 90-statement-questionnaire to evaluate five schema domains and 18 schemas after adding recognition seeking, punitiveness, and negativity/pessimism. The 18 schemas are Emotional Deprivation, Abandonment/Instability, Mistrust/Abuse, Defectiveness/Shame, Social Isolation/Alienation, Dependence/Incompetence, Vulnerability to Harm or Illness, Enmeshment/Undeveloped Self, Failure, Entitlement/Grandiosity, Insufficient Self-Control/Self-Discipline, Subjugation, Self-Sacrifice, Emotional Inhibition, Unrelenting Standards/Hypercriticalness, Approval-Seeking/Recognition-Seeking, Negativity/Pessimism, and Punitiveness (Table 2).

**Table 2:** Sociodemographic characteristics of participants.

Parameter	Migraine group (n = 50)	Tension-type headache group (n = 50)	Control group (n = 50)	*P* value
Age, years	36.3 ± 9.1	39.1 ± 7.5	37.6 ± 9.3	> 0.005
Working status				
Employed (n, %)	23 (46%)	29 (58%)	24 (48%)	0.437
Marital status				
Single/divorced (n, %)	18 (36%)	8 (16%)	13 (26%)	0.074
Educational degree				
Middle school (n, %)	15 (30%)	18 (36%)	16 (32%)	0.950
High school (n, %)	15 (30%)	13 (26%)	16 (32%)	
University or higher (n, %)	20 (40%)	19 (38%)	18 (36%)	
Financial status				
Low income (n, %)	7 (14%)	1 (2%)	6 (12%)	0.266
Middle income (n, %)	32 (64%)	37 (74%)	31 (62%)	
High income (n, %)	11 (22%)	12 (24%)	13 (26%)	
Previous psychiatric diagnosis				
Anxiety disorder (n, %)	2 (4%)	15 (30%)	7 (14%)	< 0.001
Depression (n, %)	17 (34%)	26 (52%)	21 (42%)	
Psychiatric drug usage, (n, %)	19 (38%)	41 (82%)	28 (56%)	< 0.001

The Turkish version of the YSQ-S3^24^ is composed of 90 questions using a Likert scale ranging from.

1 to 6:

- 1 = completely false, that is, definitely has nothing to do with what happens to me
- 2 = false most of the time; that is, it has almost nothing to do with what happens to me
- 3 = a little truer than false; that is, it has little to do with what happens to me
- 4 = moderately true; that is, in some way, it has something to do with what happens to me
- 5 = true most of the time; that is, it has a lot to do with what happens to me
- 6 = describes me perfectly; that is, it has everything to do with what happens to me.

There were no cut-off values for the questionnaire. High scores for specific statements indicate the existence and severity of the related EMS.

### Statistical analysis

All statistical analyses were performed using the IBM SPSS Statistics for Windows (version 21.0; IBM Corp., Armonk, NY, USA). The distribution of continuous variables was tested using the Kolmogorov–Smirnov test. Accordingly, One-way ANOVA was used to compare the continuous variables according to the distribution of the data. Chi-square or Fisher’s exact test was used to compare categorical variables. Continuous variables were presented as mean ± SD whereas categorical variables were presented as counts and percentages. We calculated the minimum number of individuals to be sampled with 95% power and 0.05 Type-I error of at least 90 (R 3.0.1. open-source program).

## Results

The sociodemographic characteristics of all the three groups were similar in terms of mean age, marital status, economic income, employment status, and degree of literacy (Table 2). However, there were significant differences in previous psychiatric diagnoses and drug use. When we compared YSQ scores, the groups differed in many schema categories (Table 3). The migraine group showed significantly higher scores in dependency/incompetency, unrelenting standards, and punitiveness schemas than the remaining two groups. The tension-type headache group had significantly more points for emotional deprivation, vulnerability to harm or illness schemas among all groups. On the other hand, the control group had significantly higher scores for insufficient self-discipline and entitlement/grandiosity schemas than the migraine and tension-type headache groups. Other differences between the groups are presented in Table 3.

**Table 3:** Comparison of Young Schema Scale scores of all groups.

Schema type	MH group mean score (n = 50)	TTH group mean score (n = 50)	C group mean score (n = 50)	${\eta }^{2}$	MH vs TTH	MH versus C	TTH versus C
Emotional deprivation	10.5 ± 4.5	16.2 ± 6.5	10.3 ± 4.4	0.245	< 0.001*	0.993	< 0.001*
Fear of abandonment	12.5 ± 5.6	12.4 ± 5.4	12.2 ± 5.3	0.001	1	1	1
Mistrust	11.2 ± 4.8	11.2 ± 4.2	11.6 ± 5.0	0.002	1	1	1
Social isolation/alienation	10.2 ± 3.8	13.7 ± 6.2	10.8 ± 4.7	0.111	0.003*	0.88	0.027
Defectiveness/shame	11.5 ± 5.4	11.4 ± 4.7	12.0 ± 5.9	0.002	1	1	1
Failure	11.3 ± 4.6	12.7 ± 5.4	11.8 ± 5.2	0.013	0.539	1	1
Dependency/incompetency	13.5 ± 7.4	12.5 ± 6.5	8.7 ± 3.4	0.226	0.85	< 0.001*	0.001*
Vulnerability to harm or illness	10.8 ± 3.6	14.0 ± 6.4	9.9 ± 4.1	0.131	0.009*	0.619	0.001*
Enmeshment	13.5 ± 6.9	13.9 ± 7.5	9.9 ± 3.9	0.163	0.995	0.005*	0.004*
Subjugation	12.0 ± 7.0	13.6 ± 5.5	12.2 ± 5.3	0.013	0.598	1	0.748
Self-sacrifice	12.6 ± 6.0	12.7 ± 6.7	9.6 ± 3.3	0.141	1	0.009*	0.015*
Emotional inhibition	11.7 ± 5.2	12.6 ± 6.3	10.5 ± 3.9	0.043	0.826	0.511	0.154
Unrelenting standards	14.3 ± 7.1	10.5 ± 3.9	9.5 ± 3.2	0.168	0.005*	< 0.001*	0.417
Entitlement/grandiosity	10.1 ± 3.2	10.2 ± 3.3	14.2 ± 8.1	0.112	0.997	0.004*	0.006*
Insufficient self-discipline	10.0 ± 3.3	10.0 ± 3.8	13.7 ± 7.5	0.104	0.999	0.008*	0.008*
Recognition-seeking	11.1 ± 4.4	10.9 ± 4.6	10.8 ± 4.4	0.001	0.994	0.994	1
Negativity/pessimism	11.4 ± 5.0	15.5 ± 8.3	9.9 ± 3.3	0.185	0.002*	0.651	< 0.001*
Punitiveness	16.8 ± 8.2	12.8 ± 4.7	10.8 ± 4.2	0.188	0.012*	< 0.001*	0.08

${\eta }^{2}$: Partial eta-squared, 0.01–0.06: Sample effect size, 0.06–0.14: Medium effect size, 0.14 or higher: Large effect size.

*MH* migraine headache, *TTH* tension-type headache, *C* control.

*p value is significant for < 0.017 due to heterogeneity of variances. Significant values are in bold.

## Discussion

In the present study, we found that the presence of early maladaptive schemas in patients with headaches differed significantly from that in the control group. In addition, the types of early maladaptive schemas were significantly different between female patients with migraine and those with tension-type headache among this whole headache. The migraine group exhibited significantly more unrelenting standards/hypercriticalness, entitlement/grandiosity, and punitiveness schemas than did the tension-type headache and control groups. Nonetheless, the tension-type headache group had significantly higher scores for negativity/pessimism, emotional inhibition, social isolation and alienation, and vulnerability to harm or illness than the migraine and control groups. Patients in both migraine and tension-type headache groups had similar dependence/incompetence, enmeshment/undeveloped self, self-sacrifice, and insufficient self-control/self-discipline schemas, although they were significantly higher than those in the control group.

Looking from past to present, it is undeniable that inequality between men and women has a negative impact on women's social lives. Young established his schema theory on the basis that a person who has had toxic experiences in childhood has a significant impact on his/her self, other, and perceptions of the world. Early maladaptive schemas derive their origin from the interaction between previous experiences and temperament. In this context, it is clear that gender roles and culture have a significant impact on schema formation. Especially during the period when sexual identity is formed, the gender adopted by significant others and the gender role that the individual perceives from the significant other are very effective on self-perception and personality traits. Especially in our culture, the importance of EMS and psychosomatic diseases in the psychological treatment of women has led us to investigate diseases that are resistant and tend to become chronic, such as migraine and tension headache, which reduce functionality.

Screenings and research conducted around the world indicate that a significant proportion of patients applying to physicians from various branches, especially internal medicine specialists and emergency departments, are psychosomatic patients. This rate increases even more in developing countries. Psychosomatic disorders are the emotional and intellectual conflicts in our lives and inner world that manifest themselves as physical symptoms, physical complaints and diseases. Person; It is a complex whole with biological, psychological, social and cultural dimensions. All these dimensions are in both health and balance; they are in constant interaction with each other in both diseases and problems. Psychosomatic diseases and disorders in which psychological factors have a significant influence, such as chronic headaches, it is the most obvious example of this mutual interaction between the soul and the body, in other words, the mutual interaction between our emotions, thoughts and body. Lipowski^20^ stated that somatization is an unresolved problem that has been known for a long time and is very commonly seen in the distinction between mental and physical medicine. Somatization is defined as a physical response to psychosocial stress and the resulting behavior of seeking medical help. Recently, Barsky et al.^21^ proposed the view of exaggerating bodily sensations (somatosensory amplification) as the central predisposing factor to explain somatization. According to this theory, individuals who somatize tend to perceive normal bodily sensations as intensely harmful and disturbing. It has been suggested that this situation is related to the somatization process^22^. Somatosensory amplification occurs in three ways: first, a state of increased attention and arousal to bodily sensation. Second, selective concentration on some weak and rare cases. Finally, reacting to bodily sensations, affects, and cognitions that make them more uncomfortable and threatening. Taken together, primary headaches are seen as a neurological pathology, but their relationship with psychiatric disorders and psychopathological processes should also be frequently emphasized.

In the current literature, no studies have sought to investigate migraine, tension-type headache, and control groups separately in terms of EMS. However, some studies have been conducted on chronic pain, somatization, depression, and anxiety. These studies showed that migraine and tension-type headaches are generally positively correlated with all EMS. In our study, the most common EMS in both headache groups were enmeshment/undeveloped self-and dependence/incompetence schemas. Similar to our findings, these two schemas are highly related to chronic pain^15^, depression and anxiety^17^, and somatization^18^. People who had high scores for the enmeshment/undeveloped self usually used somatization to cope with feelings of insecurity and desperation, and it was thought that these feelings might surface as physical pain^16^. It has been postulated that emotional and psychological distress may result in physical pain^23^. We suggest that it is possible to propose a similar judgement for EMS.

In the tension-type headache group, we found that failure schema in the impaired autonomy and performance domains was significantly more common than in the migraine and control groups. Due to the lack of success beliefs in failure schemas, patients constantly compare themselves with others, and these feelings of failure come to the surface as physical findings and contribute to the occurrence and development of pain. In the same domain, vulnerability to harm or illness schema was significantly more common in both the migraine and tension-type headache groups than in the controls. In this schema, catastrophic feelings and avoidance behaviors appear in response to threats. Furthermore, feelings of despair and anxiety may lead to other negative emotions, making it impossible to cope with the situation. It appears that physical signs such as pain in migraine and tension-type headaches are a result of failure in coping actions. Emotional deprivation, one of the EMS in the domain of disconnection and rejection, causes negative beliefs that one’s emotional needs will never be satisfied. Likewise, social isolation and alienation in the same domain cause loneliness and feelings of isolation, which make coping difficult and result in the development of headaches. Unrelenting standards/hypercriticalness schemas were more common in both migraine and tension-type headache patients than in controls; however, it was significant only in the migraine group. Negativity/pessimism and unrelenting standards/hypercriticalness schemas are part of the hypervigilance and inhibition domains, yet they are significantly more common in both headache groups than in the controls. Patients with these schemas show feelings of setting high standards and underlying emotions of fear from rejection and unworthiness as well as negativism and anxiety. It has been suggested that these factors play a role in the occurrence of chronic headache.

Self-sacrifice EMS in “orientation to the other” domain is characteristic with heavily guilt and unworthiness feelings that create self-targeted rage. Exposure to these feelings constantly and for a long time without proper coping might be a reason for experiencing physical pain in both migraine and tension-type headache patients^12,16,23^.

Substantial data show that perfectionism, inflexible thinking, self-targeted range, and other suppressed emotions and somatization play major roles in the genesis of chronic pain^24^. Without overlooking the relationship between somatization and headache, one might suggest that emotional distress caused by beliefs comes with schemas, and difficulty in coping results in the development of somatization and pain^25^.

In this study, we found that patients with headaches (migraine or tension type) had significantly lower entitlement/grandiosity and insufficient self-control schemas. This finding is compatible with the current literature: entitlement/grandiosity and insufficient self-control schemas were negatively correlated with chronic pain, depression, somatization, and anxiety in many studies^17,26,27^. According to Young’s schema theory^13^, every EMS functions in a person’s life if its effects are strong. In the Turkish version of the Young Schema Inventory, the “impaired limits” domain is a fusion of entitlement/grandiosity and insufficient self-control schemas. Keeping this in mind, we may suggest that this schema is within normal limits, and it is useful for a healthy ego functionality by making a person feel special and important by easing self-control to some degree. From this point of view, it could be asserted that patients with migraine and tension-type headaches do not see themselves as privileged, control their impulses tightly, suppress them and do not allow flexibility, have trouble expressing their feelings, or protect their rights.

We believe that our findings are important because they provide data about critical points in the psychotherapeutic treatment of migraine and tension-type headaches. When evaluated with similar studies, it would be helpful to provide a more holistic approach for the treatment of headaches that focuses on neurological, psychological, and environmental factors. This would help to better understand headaches and contribute to therapy. Especially in diseases such as chronic headache, it would be a smarter treatment approach to focus on sources, not symptoms, similar to schema therapy advice. Our study not only revives unmet childhood needs in schema domains, such as disconnection and rejection, impaired autonomy and performance, orientation to the other, and schemas such as unrelenting standards, but also advises to struggle in these fields. On the other hand, we point out the importance of a treatment plan that would provide flexibility for entitlement/grandiosity and insufficient self-control schemas classified in the impaired limits domain. In addition, we emphasize the importance of a psychotherapeutic treatment strategy that avoids the use of constant and inappropriate drug therapy to decrease symptoms. Moreover, a parenting model that focuses on childhood needs and personality patterns, which are emphasized in schema therapy, could be advantageous in preventing chronic headache. Employing such a parenting model would reduce the health costs for these patients. Our results showed that there were concentrations in different schema domains for tension-type headache and migraine-type headache, both among themselves and compared to the control groups. Our study emphasizes the importance of disconnection/rejection and impaired autonomy/performance in tension-type headaches and overvigilance/inhibition domains in migraine-type headaches for therapists. Schema domains are signals of patients’ childhood unmet needs and inappropriate coping methods with current stressors. Therapists’ strategies for working with each patient's early maladaptive schema origins will also vary in light of these signals. The difference between schema domains will be very decisive in directing the medical treatment and psychotherapeutic approach to these patients.

Our study has some limitations. First, our data were limited to a single center, even though it is a tertiary care center for a large population. If it was a multicenter study, it would provide more comprehensive data about different demographic population. People from wider spectrum of cultures could show different EMS and be using different coping mechanisms. Second, we used the Turkish version of the Young Schema Scale in our study group. Therefore, the results we reached in our study are limited to the qualities of the data that the Turkish version of the Young Schema Scale can provide. However, it was validated in a group that mostly consisted of university students. The study and control groups that we enrolled in our study may not exactly match the demographically validated group. Third; although three groups were similar in economic status, most of our subjects had middle- or high-income; therefore, our results may be less applicable to lower-income patients. At this point, we may suggest that this limitation is also applicable to Turkish version of the Young Schema Scale which consists of mostly lower- and middle-income individuals. Last two limitations cause less-demographic diversity and relative homogeneity in our study group. Naturally, this makes our results and projections less applicable to general population. At this point, authors would like add that our findings are difficult to generalize to a certain group of people due to exclusion criteria, which are mostly related with cognitive incapability, Turkish version of the Young Schema Scale was not appliable to that population. Multicenter trials with larger populations that recruit a more balanced socioeconomic status distribution among subjects would yield more conclusive results. Fourth, because our study targeted women’s mental health, we enrolled only female gender, which is why our results are not applicable to the general population. Trials in which the authors applied schema therapy that focused on groups would reach more patients and quickly collect detailed data.

## Conclusion

Recent trials and clinical practice showed that schema therapy may play a key role in not only resistant psychiatric diseases but also psychosomatic diseases such as fibromyalgia, chronic pain and migraine. Based on the fact that psychosomatic diseases are more common in women and women seek treatment for these diseases more than men, we suggest that our study may be a precursor to future studies on schema therapy approaches in women with migraine and tension-type headache.

## Data Availability

The datasets used and/or analyzed during the current study available from the corresponding author on reasonable request.
